# Effects of Fish Oil Supplementation on Cardiometabolic Risk Factors in Overweight or Obese Children and Adolescents: A Meta-Analysis of Randomized Controlled Trials

**DOI:** 10.3389/fped.2021.604469

**Published:** 2021-04-27

**Authors:** Shaojing Wu, Chunhong Zhu, Zhen Wang, Shumei Wang, Pengfei Yuan, Tao Song, Xiaoli Hou, Zhixian Lei

**Affiliations:** ^1^Department of Clinical Nutrition, Hainan Maternal and Children's Medical Center, Haikou, China; ^2^Department of Pediatrics, Zhongshan Hospital Affiliated to Dalian University, Dalian, China; ^3^Department of Critical Medicine, Hainan Maternal and Children's Medical Center, Haikou, China

**Keywords:** fish oil, eicosapentaenoic acid, docosahexaenoic acid, children, adolescents, obesity

## Abstract

**Background:** Influences of fish oil supplementation on body weight and other cardiometabolic factors in overweight or obese children and adolescents remain not fully understood. We performed a systematic review and meta-analysis of randomized controlled trials (RCTs) to evaluate the role of fish oil for these children.

**Methods:** Relevant studies were obtained by search of PubMed, Embase, and Cochrane's Library databases. A random-effect model, which incorporates the potential heterogeneity of the included studies, was used to pool the results.

**Results:** Twelve RCTs including 1,028 overweight or obese children and adolescents were included. Compared to control, fish oil supplementation significantly reduced body mass index [BMI, mean difference (MD): −0.96 kg/m^2^, 95% confidence interval (CI): −1.69 to −0.23, *P* = 0.01] but did not significantly reduce body weight or waist circumference (*P* = 0.68 and 0.76). Moreover, fish oil supplementation significantly reduced serum triglyceride (MD: −0.24 mmol/L, 95% CI: −0.40 to −0.08, *P* = 0.004) but did not significantly affect serum total cholesterol and high-density or low-density lipoprotein cholesterol (*P* = 0.83, 0.42, and 0.31, respectively). Additionally, fish oil supplementation significantly lowered systolic blood pressure (SBP, MD: −2.46 mmHg, 95% CI: −4.93 to −0.01, *P* = 0.04) but did not significantly change diastolic blood pressure (*P* = 0.22). Supplementation with fish oil did not significantly affect fasting plasma glucose (*P* = 0.33).

**Conclusions:** In overweight or obese children and adolescents, supplementation with fish oil could reduce BMI, decrease serum triglyceride, and lower SBP, while serum cholesterol and fasting glucose may not be significantly affected.

## Introduction

Obesity in children and adolescents has become an important public health problem (1, 2). A recent systematic review and meta-analysis of 103 studies including 477,620 children aged 2 to 13 years from 28 countries showed a prevalence of combined prevalence of overweight and obesity of up to 30% in 2016 (3). In the United States, about 10% of adolescents had severe obesity according to the NHANES 2013–2014 data (4). Moreover, a considerable increase in Class I obesity has been noticed in children of 2 to 5 years old in the 2015–2016 NHANES cycle (4). Childhood obesity has been associated with comorbidities of almost every body system (2). Notably, obesity in children and adolescents has been associated with increased prevalence of various cardiometabolic disorders, such as dyslipidemia, hypertension, diabetes, metabolic syndrome, etc., which could finally contribute to increased risk of adult cardiovascular diseases and mortality (5–7). Therefore, interventions targeting obesity and related cardiometabolic risk factors in overweight or obese children and adolescents are important to improve their lifetime health status (8, 9).

Nutritional intervention has become an important component of lifestyle intervention in this population (10). Fish oil, which mainly consists of the marine omega-3 polyunsaturated fatty acids (n-3 PUFAs) eicosapentaenoic acid (EPA) and docosahexaenoic acid (DHA), has been suggested to reduce weight in adults (11–13). However, previous randomized controlled trials (RCTs) evaluating the efficacy of fish oil supplementation on body weight in overweight or obese children and adolescents showed inconsistent results (14–25). Moreover, fish oil supplementation has been associated with other benefits in obese adults, such as lowering of blood pressure (BP) (26) and improving dyslipidemia (27). However, whether fish oil supplementation in overweight or obese children and adolescents also confers similar benefits on cardiometabolic risk factors including BP, lipid profile, etc. remains unknown. Therefore, in this study, we performed a meta-analysis of RCTs to evaluate the influences of fish oil on childhood obesity and related cardiometabolic risk factors.

## Methods

The PRISMA (Preferred Reporting Items for Systematic Reviews and Meta-Analyses) statement (28) and the Cochrane Handbook guidelines (29) were followed during the designing and implementation of the study.

### Search Strategy

PubMed, Embase, and the Cochrane Library (Cochrane Center Register of Controlled Trials) databases were searched for relevant studies with a combined strategy of: (1) “omega-3 fatty acids” OR “fish oil” OR fish-oil OR “polyunsaturated fatty acids” OR “marine oil” OR “eicosapentaenoic acid” OR “docosahexaenoic acid” OR “DHA” OR “EPA”; (2) “child” OR “children” OR “adolescent” OR “pediatric” OR “pediatric”; (3) “obese” OR “obesity” OR “overweight”; AND (4) “random” OR “randomly” OR “randomized” OR “randomized.” Only clinical studies published in English or Chinese were considered. The references of related reviews and original articles were also searched as a complementation. The latest database search was conducted on June 25, 2020.

### Study Selection

Inclusion criteria were as follows: (1) peer-reviewed articles in English or Chinese; (2) designed as crossover or parallel-group RCTs; (3) included overweight or obese children AND adolescents who were randomly allocated to an intervention group with fish oil supplementation or a control group with placebo or no treatment; and (4) reported at least one of the following outcomes, including changes of body weight, body mass index (BMI), waist circumference, blood lipids [triglyceride (TG), total cholesterol (TC), high-density lipoprotein cholesterol (HDLC), and low-density lipoprotein cholesterol (LDL-C)], systolic or diastolic blood pressure (SBP or DBP), and fasting plasma glucose (FPG). Reviews, studies including adults, preclinical studies, observational studies, and repeated reports were excluded.

### Data Extraction and Quality Assessment

Study search, data extraction, and quality evaluation were achieved by two independent authors. If disagreement occurred, it was resolved by consensus between the two authors. We extracted data regarding study information (first author, publication year, and study country), study design (blind or open-label, crossover, or parallel group), participant characteristics (number of participants, mean age, gender, and health status), regimens of fish oil and controls, and treatment durations. Quality evaluation was achieved using the Cochrane's Risk of Bias Tool (29) according to the following aspects: (1) random sequence generation, (2) allocation concealment, (3) blinding of participants and personnel, (4) blinding of outcome assessors, (5) incomplete outcome data, (6) selective outcome reporting, and (7) other potential bias.

### Statistical Analysis

All endpoints were estimated based on the change from baseline to follow-up, and pooled effects were presented as mean differences (MDs) with 95% confidence interval (CI). We used the Cochrane's *Q* test to detect the heterogeneity, and significant heterogeneity was suggested if *P* < 0.10 (30). The *I*^2^ statistic was also calculated, and an *I*^2^ > 50% reflected significant heterogeneity. In view of the clinical heterogeneity among the included studies regarding the characteristics of participants and interventions, a random-effect model, which is considered as a conservative method by incorporating the heterogeneity among the included studies, was applied to pool the results (29). Subgroup analyses were performed to evaluate the influences of study characteristics on the outcomes, including study design, health status of the participants, dose of fish oil, and treatment durations. Medians of the continuous variables were chosen as the cutoff value for defining subgroups. Publication bias was evaluated by visual inspection of funnel plots and Egger's regression asymmetry test (31). *P*-values < 0.05 were considered statistically significant. The RevMan (Version 5.1; Cochrane, Oxford, UK) and Stata software (Version 12.0; Stata, College Station, TX) were applied for statistical analyses.

## Results

### Search Results

**Figure 1:**
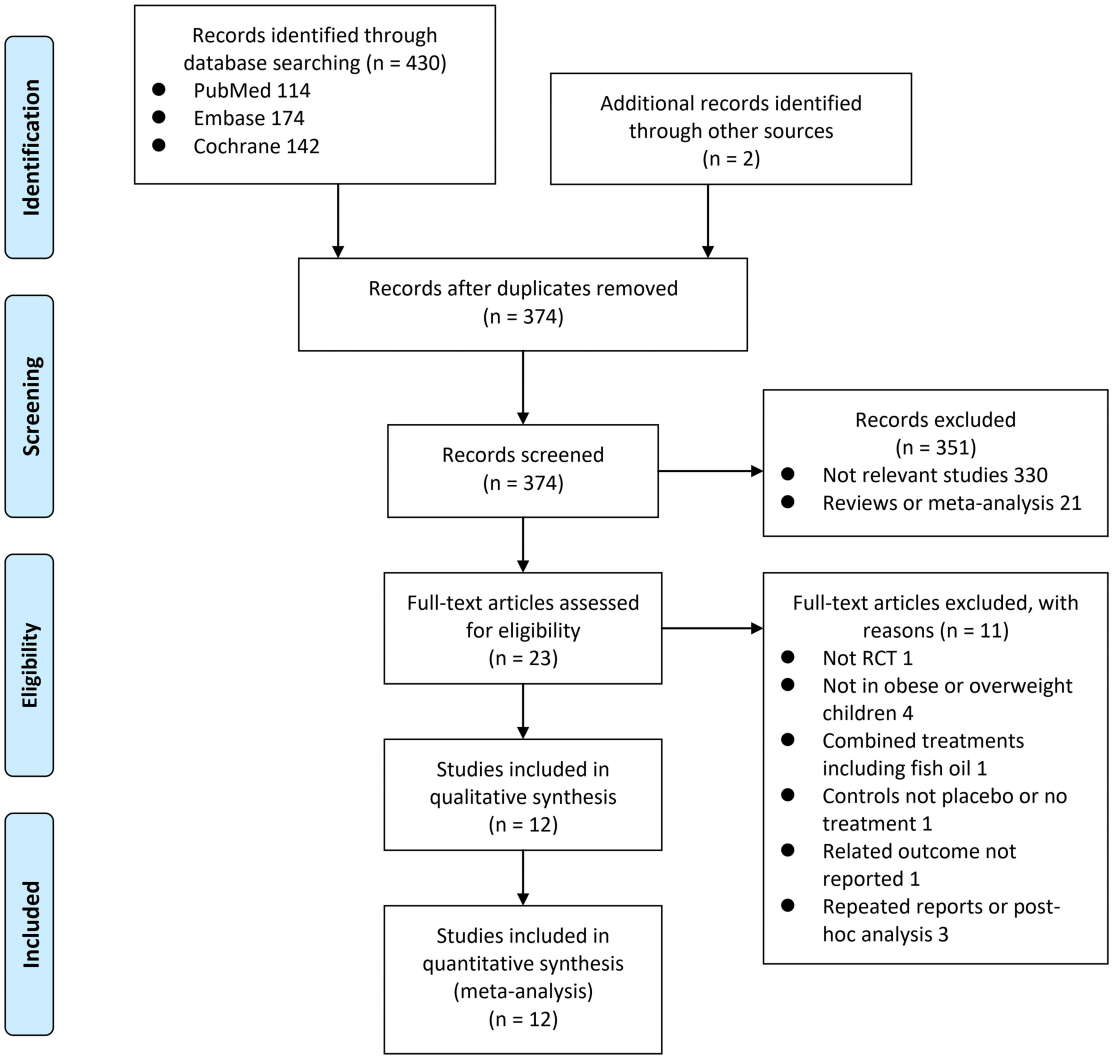
Flowchart of literature search.

In summary, 432 studies were obtained through the initial database search. After exclusion of duplicate studies, 374 studies were screened. Among them, 351 studies were subsequently excluded based on titles and abstracts primarily because these studies were not irrelevant. Among the 23 potentially relevant articles, 11 were further excluded via full-text review based on reasons listed in Figure 1. Finally, 12 RCTs were included in the meta-analysis (14–25).

### Study Characteristics and Quality Evaluation

Table 1 shows the characteristics of the included studies. Overall, 12 RCTs including 1,028 overweight or obese children and adolescents were included. These studies were published between 2010 and 2019 and performed in Sweden (14), Denmark (15), Mexico (16, 23–25), the Czech Republic (17), the United States (18, 19), Turkey (20), Poland (21), and Italy (22). Three studies were crossover RCTs (14, 17, 19), while the remaining RCTs were of parallel-group design. All the RCTs included overweight or obese children and adolescents, among which four studies included those with hypertriglyceridemia (18, 19, 23, 24), and the other three included those with non-alcoholic fatty liver disease (NAFLD) (20–22). The mean ages of the included children and adolescents varied between 10 and 16 years. The total dose of fish oil varied from 250 to 3,000 mg/day, with EPA and DHA ranging within 0–2,000 and 177–1,500 mg/day, respectively. The treatment durations varied from 3 to 52 weeks. Table 2 shows the details of study quality evaluation. All of the included RCTs were double blinded except for two studies (16, 17). Methods of random sequence generation were reported in two studies (16, 25), and information of allocation concealment was reported in only one study (25). The overall quality score varied within 3 to 6.

**Table 1:** Characteristics of the included RCTs.

**Study**	**Country**	**Study**	**Sample**	**Healthy**	**Mean age**	**Male**	**Fish oil dose**	**EPA**	**DHA**	**Control**	**Treatment duration**
		**design**	**size**	**status**							
					**years**	**%**	**mg/day**	**mg/day**	**mg/day**		**weeks**
Dangardt et al. (14)	Sweden	R, DB, PC, CO	25	Obese adolescents	15.7	44	1,220	930	290	Medium-chain triglycerides	12
Pedersen et al. (15)	Denmark	R, DB, PC	78	Slightly overweight adolescent boys	14.3	100	1,500	400	1,100	Vegetable oil	16
Lopez-Alarcon et al. (16)	Mexico	R, PC	76	Obese prepubertal and pubertal children	13.4	NR	900	540	360	Corn starch	4
Vasickova et al. (17)	Czech	R, CO	120	Obese children	10.0	NR	340	42	300	No treatment	3
De Ferranti et al. (18)	USA	R, DB, PC	24	Overweight or obese adolescents with hypertriglyceridemia	14.0	58	3,360	1,860	1,500	Corn oil	26
Gidding et al. (19)	USA	R, DB, PC, CO	42	Obese adolescents with hypertriglyceridemia	14.0	69	3,360	1,860	1,500	Corn oil	8
Boyraz et al. (20)	Turkey	R, DB, PC	108	Obese adolescents with NAFLD	13.8	51	1,000	560	440	NR	52
Janczyk et al. (21)	Poland	R, DB, PC	64	Overweight/obese children with NAFLD	13.0	85	450, 900, and 1,300 for BW < 40, 40–60, and >60 kg	267, 534, and 800 for BW < 40, 40–60, and >60 kg	177, 355, and 500 for BW < 40, 40–60, and >60 kg	Sunflower oil	26
Pacifico et al. (22)	Italy	R, DB, PC	51	Overweight children with NAFLD	10.9	59	250	0	250	Germ oil	26
Huang et al. (24)	Mexico	R, DB, PC	65	Obese adolescents with hypertriglyceridemia	12.5	NR	3,000	2,000	1,000	Soybean oil	12
Del-Rio-Navarro et al. (23)	Mexico	R, DB, PC	130	Obese children with hypertriglyceridemia	12.6	62	3,000	2,000	1,000	Soybean oil	12
Lopez-Alarcon et al. (25)	Mexico	R, DB, PC	245	Pubertal children with obesity	13.6	48	1,200	800	400	Sunflower oil	12

*RCT, randomized controlled trials; R, randomized; DB, double-blinded; PC, placebo-controlled; CO, crossover; NAFLD, non-alcoholic fatty liver disease; BW, body weight; NR, not reported; EPA, eicosapentaenoic acid; DHA, docosahexaenoic acid*.

**Table 2:** Quality evaluation of the included studies via the Cochrane's Risk of Bias Tool.

**Study**	**Random sequence generation**	**Allocation concealment**	**Blinding of participants**	**Blinding of outcome assessment**	**Incomplete outcome data addressed**	**Selective reporting**	**Other sources of bias**	**Total**
Dangardt et al. (14)	Unclear	Unclear	Low	Low	Low	Low	Low	5
Pedersen et al. (15)	Unclear	Unclear	Low	Low	Low	Low	Unclear	4
Lopez-Alarcon et al. (16)	Low	Unclear	Unclear	Unclear	Low	Low	Low	4
Vasickova et al. (17)	Unclear	Unclear	Unclear	Unclear	Low	Low	Low	3
De Ferranti et al. (18)	Unclear	Unclear	Low	Low	Low	Low	Low	5
Gidding et al. (19)	Unclear	Unclear	Low	Low	Low	Low	Unclear	4
Boyraz et al. (20)	Unclear	Unclear	Low	Low	Low	Low	Low	5
Janczyk et al. (21)	Unclear	Unclear	Low	Low	Low	Low	Low	5
Pacifico et al. (22)	Unclear	Unclear	Low	Low	Low	Low	Low	5
Huang et al. (24)	Unclear	Unclear	Low	Low	Low	Low	Low	5
Del-Rio-Navarro et al. (23)	Unclear	Unclear	Low	Low	Low	Low	Low	5
Lopez-Alarcon et al. (25)	Low	Low	Low	Low	Low	Low	Unclear	6

### Fish Oil Supplementation on Body Weight, BMI, and Waist Circumference

Meta-analysis of seven RCTs showed that compared to control, fish oil supplementation did not significantly reduce body weight of the overweight or obese children and adolescents (MD: −0.61 kg, 95% CI: −3.84–2.26, *P* = 0.68; *I*^2^ = 0%; Table 3). However, fish oil supplementation significantly reduced BMI in these participants (MD: −0.96 kg/m^2^, 95% CI: −1.69 to −0.23, *P* = 0.01; *I*^2^ = 0%; Table 3). Pooled results of three RCTs showed that fish oil supplementation did not significantly reduce waist circumference (MD: −0.69 cm, 95% CI: −5.08–3.70, *P* = 0.76; *I*^2^ = 0%; Table 3). Subgroup analyses suggested that study characteristics such as study design, health status of the participants, dose of fish oil, or treatment durations did not significantly affect the influences of fish oil supplementation on body weight or BMI in overweight or obese children and adolescents (*P* for subgroup difference all > 0.05; Table 4).

**Table 3:** Summaries of main findings of the meta-analyses.

**Outcomes**	**No. of studies (participants)**	**Studies included**	**Main results MD (95% CI)**	***I*^**2**^**	***P* for Cochrane's *Q* test**	***P* for overall effect**
Body weight (kg)	7 (620)	(1), (4), (5), (6), (7), (9), (10)	−0.61 (−3.48–2.26)	0%	0.98	0.68
BMI (kg/m^2^)	7 (510)	(1), (5), (6), (7), (9), (10), (11)	−0.96 (−1.69 to −0.23)	0%	0.36	0.01
Waist circumference (cm)	3 (125)	(1), (5), (9)	−0.69 (−5.08–3.70)	0%	0.44	0.76
TG (mmol/L)	10 (897)	(1), (2), (5), (6), (7), (8), (9), (10), (11), (12)	−0.24 (−0.40 to −0.08)	41%	0.09	0.004
TC (mmol/L)	10 (892)	(1), (2), (4), (5), (6), (7), (8), (9), (10), (11)	0.01 (−0.12–0.15)	24%	0.22	0.83
HDL-C (mmol/L)	9 (652)	(1), (2), (5), (6), (7), (8), (9), (10), (11)	0.04 (−0.05–0.12)	41%	0.09	0.42
LDL-C (mmol/L)	6 (408)	(1), (2), (5), (6), (7), (8), (9)	0.06 (−0.05–0.17)	0%	0.82	0.31
SBP (mmHg)	7 (679)	(1), (2), (6), (7), (9), (10), (12)	−2.46 (−4.93 to −0.01)	25%	0.23	0.04
DBP (mmHg)	6 (434)	(1), (2), (6), (7), (9), (10)	−1.60 (−4.14–0.94)	61%	0.03	0.22
FPG (mmol/L)	9 (644)	(1), (2), (3), (5), (7), (8), (9), (10), (11)	0.06 (−0.06–0.18)	66%	0.003	0.33

*BMI, body mass index; TG, triglyceride; TC, total cholesterol; HDL-C, high-density lipoprotein cholesterol; LDL-C, low-density lipoprotein cholesterol; SBP, systolic blood pressure; DBP, diastolic blood pressure; FPG, fasting plasma glucose*.

*Studies included: (1) Dangardt et al. (14); (2) Pedersen et al. (15); (3) Lopez-Alarcon et al. (16); (4) Vasickova et al. (17); (5) De Ferranti et al. (18); (6) Gidding et al. (19); (7) Boyraz et al. (20); (8) Janczyk et al. (21); (9) Pacifico et al. (22); (10) Huang et al. (24); (11) Del-Rio-Navarro et al. (23); (12) Lopez-Alarcon et al. (25)*.

**Table 4:** Subgroup analyses for the outcomes of BW and BMI.

	**BW (kg)**			**BMI (kg/m**^****2****^**)**		
**Characteristics**	**No. of studies**	**MD (95% CI)**	***P***	**No. of studies**	**MD (95% CI)**	***P***
**Study design**						
Crossover	3	−0.61 [−4.74, 3.52]		2	−0.06 [−1.86, 1.74]	
Parallel	4	−0.60 [−4.59, 3.38]	1.00	5	−1.14 [−1.94, −0.33]	0.28
**Health status**						
Non-NAFLD	5	−1.17 [−4.66, 2.32]		5	−0.55 [−1.40, 0.30]	
NAFLD	2	0.56 [−4.47, 5.60]	0.58	2	−2.08 [−3.50, −0.67]	0.07
**Dose of fish oil**						
≤ 1,500 mg/day	4	−0.11 [−3.48, 3.26]		3	−1.56 [−2.77, −0.34]	
>1,500 mg/day	3	−1.91 [−7.35, 3.54]	0.58	4	−0.62 [−1.53, 0.30]	0.23
**Dose of EPA**						
≤ 1,000 mg/day	4	−0.11 [−3.48, 3.26]		3	−1.56 [−2.77, −0.34]	
>1,000 mg/day	3	−1.91 [−7.35, 3.54]	0.58	4	−0.62 [−1.53, 0.30]	0.23
**Dose of DHA**						
≤ 500 mg/day	4	−0.11 [−3.48, 3.26]		3	−1.56 [−2.77, −0.34]	
>500 mg/day	3	−1.91 [−7.35, 3.54]	0.58	4	−0.62 [−1.53, 0.30]	0.23
**Treatment durations**						
≤ 12 weeks	4	−1.24 [−4.81, 2.33]		4	−0.58 [−1.46, 0.29]	
>12 weeks	3	0.54 [−4.27, 5.35]	0.56	3	−1.80 [−3.12, −0.48]	0.13

*BW, body weight; BMI, body mass index; MD, mean difference; CI, confidence interval; NAFLD, non-alcoholic fatty liver disease; EPA, eicosapentaenoic acid; DHA, docosahexaenoic acid*.

### Fish Oil Supplementation on Blood Lipids

Meta-analysis of 10 RCTs showed that fish oil supplementation significantly reduced serum level of TG compared to control in overweight or obese children and adolescents (MD: −0.24 mmol/L, 95% CI: −0.40 to −0.08, *P* = 0.004; *I*^2^ = 41%; Table 3). Subgroup analysis showed that TG was reduced more remarkably after high-dose fish oil supplementation (fish oil > 1,500 mg/day, EPA > 1,000 mg/day, DHA > 500 mg/day) than that after low-dose fish oil supplementation (*P* for subgroup difference all < 0.05; Table 5). Compared to control, fish oil supplementation did not significantly affect serum TC (MD: 0.01 mmol/L, 95% CI: −0.12–0.15, *P* = 0.83; *I*^2^ = 24%; Table 3), HDL-C (MD: 0.04 mmol/L, 95% CI: −0.05–0.17, *P* = 0.42; *I*^2^ = 41%; Table 3), or LDL-C (MD: 0.06 mmol/L, 95% CI: −0.05–0.17, *P* = 0.31; *I*^2^ = 0%; Table 3) levels in these participants. Subgroup analysis suggested that study characteristics including study design, health status of the participants, dose of fish oil, or treatment durations did not significantly affect the influences of fish oil supplementation on TC or LDL-C (*P* for subgroup difference all > 0.05; Table 5). However, HDL-C may be increased in studies with low-dose supplementation (fish oil ≤ 1,500 mg/day, EPA ≤ 1,000 mg/day; *P* for subgroup difference < 0.05; Table 5).

**Table 5:** Subgroup analyses for the outcomes of blood lipids.

	**TG (mmol/L)**			**TC (mmol/L)**			**HDL-C (mmol/L)**			**LDL-C (mmol/L)**		
**Characteristics**	**No. of studies**	**MD (95% CI)**	***P***	**No. of studies**	**MD (95% CI)**	***P***	**No. of studies**	**MD (95% CI)**	***P***	**No. of studies**	**MD (95% CI)**	***P***
**Study design**												
Crossover	2	−0.28 [−0.57, 0.00]		3	−0.03 [−0.23, 0.18]		2	0.01 [−0.12, 0.14]		2	0.11 [−0.09, 0.30]	
Parallel	8	−0.21 [−0.34, −0.07]	0.82	7	0.04 [−0.14, 0.22]	0.63	7	0.04 [−0.07, 0.16]	0.69	4	0.03 [−0.10, 0.17]	0.55
**Health status**												
Non-NAFLD	7	−0.29 [−0.52, −0.06]		7	0.02 [−0.13, 0.17]		6	−0.01 [−0.10, 0.08]		4	0.05 [−0.08, 0.18]	
NAFLD	3	−0.14 [−0.37, 0.10]	0.35	3	0.03 [−0.33, 0.39]	0.98	3	0.13 [−0.03, 0.29]	0.14	2	0.07 [−0.13, 0.27]	0.69
**Dose of fish oil**												
≤ 1,500 mg/day	6	−0.07 [−0.22, 0.08]		6	0.06 [−0.17, 0.28]		5	0.13 [0.03, 0.23]		4	0.04 [−0.08, 0.16]	
>1,500 mg/day	4	−0.55 [−0.76, −0.33]	<0.01	4	−0.04 [−0.21, 0.13]	0.50	4	−0.05 [−0.14, 0.04]	<0.01	2	0.11 [−0.13, 0.36]	0.59
**Dose of EPA**												
≤ 1,000 mg/day	6	−0.07 [−0.22, 0.08]		6	0.06 [−0.17, 0.28]		5	0.13 [0.04, 0.23]		4	0.04 [−0.08, 0.16]	
>1,000 mg/day	4	−0.55 [−0.76, −0.33]	<0.01	4	−0.04 [−0.21, 0.13]	0.50	4	−0.05 [−0.14, 0.04]	<0.01	2	0.11 [−0.13, 0.36]	0.59
**Dose of DHA**												
≤ 500 mg/day	5	−0.08 [−0.24, 0.08]		5	−0.03 [−0.23, 0.18]		4	0.11 [−0.02, 0.24]		3	0.08 [−0.09, 0.25]	
>500 mg/day	5	−0.41 [−0.68, −0.15]	0.04	5	0.06 [−0.13, 0.24]	0.57	5	−0.01 [−0.12, 0.10]	0.18	3	0.04 [−0.10, 0.18]	0.73
**Treatment durations**												
≤ 12 weeks	5	−0.35 [−0.64, −0.07]		5	−0.06 [−0.20, 0.09]		4	−0.03 [−0.12, 0.05]		3	0.05 [−0.08, 0.18]	
>12 weeks	5	−0.12 [−0.31, 0.08]	0.18	5	0.13 [−0.14, 0.40]	0.22	5	0.10 [−0.04, 0.24]	0.10	3	0.08 [−0.12, 0.27]	0.81

*BMD, mean difference; CI, confidence interval; NAFLD, non-alcoholic fatty liver disease; EPA, eicosapentaenoic acid; DHA, docosahexaenoic acid; TG, triglyceride; TC, total cholesterol; HDL-C, high-density lipoprotein cholesterol; LDL-C, low-density lipoprotein cholesterol*.

### Fish Oil Supplementation on BP and FPG

Meta-analysis of seven RCTs showed that fish oil supplementation significantly reduced SBP compared to control in overweight or obese children and adolescents (MD: −2.46 mmHg, 95% CI: −4.93 to −0.01, *P* = 0.04; *I*^2^ = 25%; Table 3), while DBP (MD: −1.60 mmHg, 95% CI: −4.14–0.94, *P* = 0.03; *I*^2^ = 61%; Table 3) or FPG (MD: 0.06 mmol/L, 95% CI: −0.06–0.18, *P* = 0.33; *I*^2^ = 66%; Table 3) was not significantly affected. Subgroup analysis suggested that study characteristics including study design, health status of the participants, dose of fish oil, or treatment durations did not significantly affect the influences of fish oil supplementation on SBP, DBP, and FPG in these participants (*P* for subgroup difference all > 0.05; Table 6).

**Table 6:** Subgroup analyses for the outcomes of blood pressure and FPG.

	**SBP (mmHg)**			**DBP (mmHg)**			**FPG (mmol/L)**		
**Characteristics**	**No. of studies**	**MD (95% CI)**	***P***	**No. of studies**	**MD (95% CI)**	***P***	**No. of studies**	**MD (95% CI)**	***P***
**Study design**									
Crossover	2	−2.07 [−7.42, 3.28]		2	−4.09 [−12.13, 3.95]		1	0.30 [0.08, 0.52]	
Parallel	5	−2.69 [−6.41, 1.03]	0.85	4	−0.48 [−2.26, 1.29]	0.39	8	0.03 [−0.09, 0.14]	0.08
**Health status**									
Non-NAFLD	5	−1.92 [−4.34, 0.49]		4	−2.48 [−5.71, 0.76]		6	0.05 [−0.08, 0.18]	
NAFLD	2	−5.48 [−18.22, 7.26]	0.59	2	0.37 [−3.48, 4.22]	0.27	3	0.08 [−0.24, 0.39]	0.88
**Dose of fish oil**									
≤ 1,500 mg/day	5	−2.45 [−6.27, 1.36]		4	−0.28 [−2.30, 1.74]		6	0.05 [−0.13, 0.22]	
>1,500 mg/day	2	−2.81 [−7.23, 1.61]	0.90	2	−4.23 [−11.57, 3.10]	0.31	3	0.08 [−0.05, 0.20]	0.77
**Dose of EPA**									
≤ 1,000 mg/day	5	−2.45 [−6.27, 1.36]		4	−0.28 [−2.30, 1.74]		6	0.05 [−0.13, 0.22]	
>1,000 mg/day	2	−2.81 [−7.23, 1.61]	0.90	2	−4.23 [−11.57, 3.10]	0.31	3	0.08 [−0.05, 0.20]	0.77
**Dose of DHA**									
≤ 500 mg/day	4	−2.48 [−7.29, 2.32]		3	0.43 [−2.02, 2.87]	0.13	5	0.06 [−0.17, 0.30]	
>500 mg/day	3	−2.93 [−6.46, 0.61]	0.88	3	−3.26 [−7.36, 0.84]		4	0.04 [−0.05, 0.13]	0.87
**Treatment durations**									
≤ 12 weeks	4	−1.82 [−4.37, 0.73]		3	−2.81 [−7.53, 1.91]		4	0.05 [−0.16, 0.25]	
>12 weeks	3	−4.57 [−11.91, 2.77]	0.49	3	−0.40 [−3.05, 2.25]	0.38	5	0.07 [−0.09, 0.23]	0.86

*MD, mean difference; CI, confidence interval; NAFLD, non-alcoholic fatty liver disease; EPA, eicosapentaenoic acid; DHA, docosahexaenoic acid; SBP, systolic blood pressure; DBP, diastolic blood pressure; FPG, fasting plasma glucose*.

### Publication Bias

**Figure 2:**
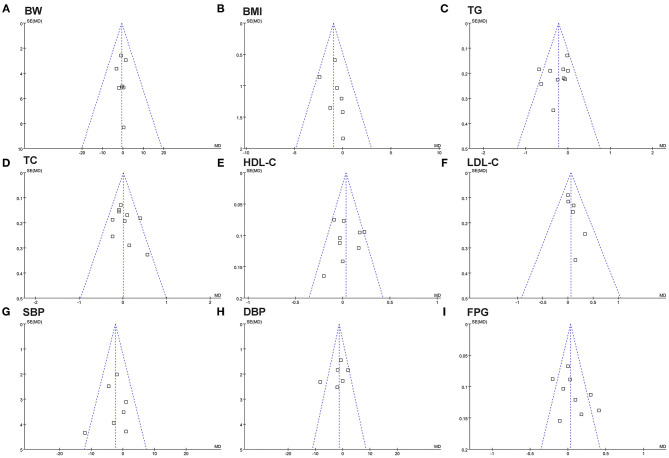
Funnel plots for the meta-analysis evaluating the influence of fish oil supplementation on obesity and other cardiometabolic risk factors in overweight or obese children. **(A)** Body weight (BW); **(B)** BMI; **(C)** TG; **(D)** TC; **(E)** HDL-C; **(F)** LDL-C; **(G)** SBP; **(H)** DBP; **(I)** FPG.

The funnel plots for the meta-analyses of the influences of fish oil supplementation on body weight, BMI, blood lipids, SBP, DBP, and FPG are shown in Figures 2A–I. The plots were symmetrical on visual inspection, suggesting low risk of publication biases. Egger's regression tests showed similar results (*P* all > 0.10). The potential publication bias underlying the meta-analysis of the influence of fish oil supplementation on waist circumference was undetermined since only three studies were included.

## Discussion

In this systematic review and meta-analysis, by pooling the results of available RCTs, we found that fish oil supplementation significantly reduced BMI in overweight or obese children and adolescents, although body weight and waist circumference was not significantly affected. Moreover, fish oil significantly decreased serum TG in these participants, and the efficacy was more remarkable in studies with higher dose of fish oil supplementation. Serum TC, HDL-C, or LDL-C was not significantly affected. In addition, fish oil supplementation was associated with a moderate BP lowering efficacy, as evidenced by a reduction of SBP by 2.42 mmHg. Supplementation with fish oil was not associated with a significant change of FPG. Taken together, these results indicated that in overweight or obese children and adolescents, fish oil supplementation could significantly reduce BMI, decrease TG, and moderately lower SBP compared to controls, suggesting a potential beneficial influence of fish oil on cardiometabolic risk factors in these participants.

To the best of our knowledge, our meta-analysis is the first meta-analysis regarding the influences of fish oil supplementation on obesity and other cardiometabolic risk factors in overweight or obese children and adolescents. One important finding of the study was that fish oil supplementation significantly reduced BMI as compared with controls in overweight or obese children and adolescents. Further subgroup analysis showed that the effect of fish oil supplementation on BMI in these participants was independent of study characteristics such as study design, health status of the participants, dose of fish oil, or treatment durations, which further confirmed the robustness of the finding. Although fish oil was not shown to reduce body weight or waist circumference significantly in this meta-analysis, in view of the superiority of BMI in diagnosis of pediatric obesity to the other two parameters (32), our results confirmed a potential anti-obese efficacy of fish oil in these participants. Previous meta-analysis in obese adults also showed that fish oil supplementation significantly reduced BMI as compared with controls (11). An early cross-sectional study in children between 5 and 12 years from Australia showed that lower omega-3 index evidenced by the erythrocyte fatty acid composition was associated with increased BMI and obesity, as well as insulin resistance (33), indicating a potential inverse association between body long-chain omega-3 polyunsaturated fatty acid (n-3 PUFA) contents and risk of obesity. A subsequent study in subcutaneous adipose tissues of obese adolescents showed that fish oil supplementation modulated the expressions of genes related to lipid metabolism, oxidative stress, and hypoxia, including PPARa, SREBP1, and PGC-1a, accompanied by reduced BMI in these participants. These results suggested that fish oil supplementation may exert its anti-obese efficacy via directly modulating the genes involved in lipid metabolism in obese adolescents (34). Our meta-analysis did not show a lowering efficacy of fish oil supplementation on body weight or waist circumference in overweight or obese children and adolescents, which was not consistent with the findings in a previous meta-analysis in obese adults (11), which may be explained by potential differences between children and adults in terms of body composition and lipid metabolism (35). Besides, for the outcome of waist circumference, only three RCTs with 125 children were included. The influences of fish oil supplementation on waist circumference in overweight or obese children and adolescents should be validated in large-scale RCTs.

As for the influences of fish oil supplementation on blood lipids, our meta-analysis showed that fish oil significantly decreased serum TG in overweight or obese children and adolescents, and the efficacy was more remarkable in studies with higher dose of fish oil supplementation. However, serum TC, HDL-C, or LDL-C was not significantly affected. The TG-lowering efficacy of fish oil in obese children was consistent with the findings of previous studies in adult patients with various clinical conditions, such as diabetes (36) and end-stage kidney diseases (37, 38). Besides, results of subgroup analyses showed that HDL-C in obese adolescents may be increased in studies with low-dose supplementation (fish oil ≤ 1,500 mg/day, EPA ≤ 1,000 mg/day) and in studies with longer treatment durations (>12 weeks), which has also been observed in previous studies including adult participants (36–38). In view of the role of HDL-C in attenuating atherosclerosis, these findings may highlight other mechanisms underlying the potential cardiometabolic benefits of fish oil in overweight or obese children and adolescents.

In addition, fish oil supplementation was found to moderately lower SBP in the overweight or obese children and adolescents. An early study has confirmed that high n-3 PUFA levels in thin/normal weight children are associated with lower and therefore healthier BP (39), suggesting a potential role of n-3 PUFA in maintaining healthy BP. Besides, endothelial dysfunction has been considered as an initial factor for hypertension in children and adults (40). Fish oil supplementation has been shown to improve endothelial function in children (24, 41), which was consistent with the findings in adult populations (42). Moreover, our meta-analysis did not show a significant influence of fish oil supplementation on FPG in obese adolescents, which is consistent with previous findings in adult patients (36).

The strengths of our meta-analysis may include the following. Firstly, only RCTs were included, which minimized the possible biases caused by studies with other designs. Secondly, only RCTs with interventions of exclusive fish oil supplementation were included, which eliminated the potential confounding effects of other co-interventions. Thirdly, the age range of the participants of the included RCTs was not wide and was representative of school-age children and adolescent populations, which ensured the homogeneity of the study population. Besides, our study also has some limitations. First, characteristics of participants and fish oil treatment regimens were varied among the included studies, such as the age, sex, and health status of the children and adolescents, as well as the dosages and treatment durations of fish oil, which may contribute to the heterogeneity among the included studies. Secondly, although subgroup analyses were performed to explore the potential influences of variances in study characteristics on the outcome, results of subgroup analyses should be interpreted with caution because limited datasets were included for each subgroup. Thirdly, the dietary intake of fish and omega-3 fatty acids were generally not reported in the included studies. Differences in the dietary omega-3 fatty acid intake may affect the potential benefits of fish oil supplementation in these participants. Finally, clinical outcomes were not evaluated in this study. Whether fish oil supplementation could reduce morbidity and mortality in overweight or obese children and adolescents may be investigated in large-scale RCTs.

In conclusion, supplementation with fish oil could reduce BMI, decrease serum triglyceride, and lower SBP in overweight or obese children and adolescents, while serum cholesterol and fasting glucose may not be significantly affected. Findings of the meta-analysis should be validated in large-scale RCTs. Moreover, studies are warranted to evaluate the influences of fish oil supplementation on lifetime morbidity and mortality in overweight or obese children and adolescents.

## Data Availability Statement

The original contributions generated for this study are included in the article/supplementary material, further inquiries can be directed to the corresponding author/s.

## Author Contributions

SWu, CZ, XH, and ZL designed the study. SWu, CZ, and ZW performed database search, literature identification, study quality evaluation, and data extraction. SWa, PY, and TS performed statistical analysis. CZ, SWa, PY, and TS interpreted the results. SWu, CZ, XH, and ZL drafted the manuscript. All authors critically revised the manuscript and approved its submission.

## Conflict of Interest

The authors declare that the research was conducted in the absence of any commercial or financial relationships that could be construed as a potential conflict of interest.
